# Evaluation of myocardial function after primary percutaneous intervention by cardial MRI: 5 years follow up

**DOI:** 10.1186/1532-429X-11-S1-P86

**Published:** 2009-01-28

**Authors:** Tirza Springeling, Sharon W Kirschbaum, Timo Baks, Yusuf Karamermer, Gabriel K Krestin, Pim J de Feyter, Robert-Jan M van Geuns

**Affiliations:** 1 Erasmus MC, Rotterdam, Netherlands; 2 Erasmus MC, Thoraxcenter, Rotterdam, Netherlands

**Keywords:** Myocardial Infarction, Percutaneous Coronary Intervention, Left Ventricular Ejection Fraction, Acute Myocardial Infarction, Late Effect

## Background

We investigated the early and late effect of primary percutaneous coronary interventions (PCI) for acute myocardial infarction on recovery of left ventricular ejection fraction (LVEF), end diastolic volume (EDV), end systolic volume (ESV) and segmental wall thickening (SWT) using Cardiac MRI.

## Methods

This abstract includes preliminary analysis of the first 12 patients. All patients underwent cardial MRI within 5 days, at 5 months and at 5 years after successful primary PCI. LVEF, EDV, ESV and SWT quantified on cine-images, the transmural extent of the infarction (TEI) was quantified on delayed-enhancement images.

## Results

**Figure 1:**
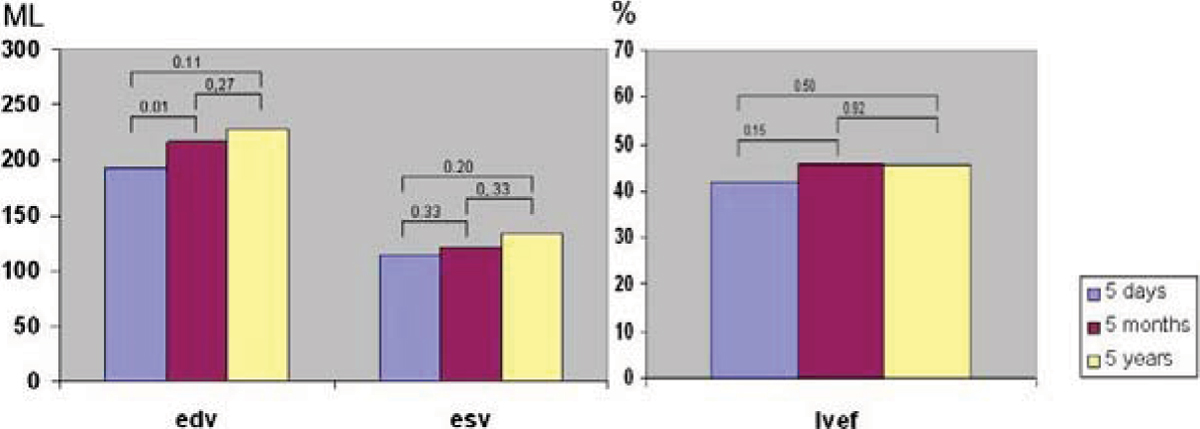
Figure 1

EDV increased significantly between baseline and 5 months (from 193.5 ± 45.4 to 216.4 ± 61.5, p = .01), with a trend for additional late remodelling at 5 years (from 216.4 ± 61.5 to 218.0 ± 82.1, p = .27). The same trend seems to be visible in ESV although also not significant (resp.114,2 ± 33.9; 122.0 ± 51.8; 132.5 ± 73.4 p = 0.20). LVEF showed some recovery between baseline and 5 months (from 41.60 ± to 45,8 ± 12.4, p = .15), with no further change after 5 months (45,8 ± 12.4; 45.56 ± 10.7 p = .92) (see figure 1).

SWT improved significantly between baseline and 5 months follow up (from 23% to 30%, p = 0.04), no additional improvement was seen at 5 years (SWT 33%, p = 0.29). TEI at baseline showed a good correlation with SWT at 5 months and 5 years follow up.

## Conclusion

These interesting preliminary results of 12 patients indicates a tendency to late remodelling at 5 years follow up by which LVEF is preserved. TEI at baseline predicted regional SWT at follow up.

